# Maternal interchromosomal insertional translocation leading to 1q43-q44 deletion and duplication in two siblings

**DOI:** 10.1186/s13039-018-0371-7

**Published:** 2018-04-04

**Authors:** Aixiang Luo, Dehua Cheng, Shimin Yuan, Haiyu Li, Juan Du, Yang Zhang, Chuanchun Yang, Ge Lin, Wenyong Zhang, Yue-Qiu Tan

**Affiliations:** 1 Institute of Reproduction and Stem Cell Engineering, Xiangya School of Medicine, Central South University, Changsha, Hunan 410078 People’s Republic of China; 2 Reproductive and Genetic Hospital of CITIC-Xiangya, Changsha, Hunan 410078 People’s Republic of China; 3 School of Biological Sciences, Faculty of Science, The University of Hong Kong, Hong Kong, 999077 People’s Republic of China; 4 Cheerland Precision Biomed Co., Ltd., Shenzhen, Guangdong 518055 People’s Republic of China; 5 Southern University of Science and Technology, Shenzhen, Guangdong 518055 People’s Republic of China

**Keywords:** Pure 1q43-q44 deletion/duplication, Insertional translocation, Congenital anomaly, Whole-genome sequencing

## Abstract

**Background:**

1q43-q44 deletion syndrome is a well-defined chromosomal disorder which is characterized by moderate to severe mental retardation, and variable but characteristic facial features determined by the size of the segment and the number of genes involved. However, patients with 1q43-q44 duplication with a clinical phenotype comparable to that of 1q43-q44 deletion are rarely reported. Moreover, pure 1q43-q44 deletions and duplications derived from balanced insertional translocation within the same family with precisely identified breakpoints have not been reported.

**Case presentation:**

The proband is a 6-year-old girl with profound developmental delay, mental retardation, microcephaly, epilepsy, agenesis of the corpus callosum and hearing impairment. Her younger brother is a 3-month-old boy with macrocephaly and mild developmental delay in gross motor functions. G-banding analysis of the subjects at the 400-band level did not reveal any subtle structural changes in their karyotypes. However, single-nucleotide polymorphism (SNP) array analysis showed a deletion and a duplication of approximately 6.0 Mb at 1q43-q44 in the proband and her younger brother, respectively. The Levicare analysis pipeline of whole-genome sequencing (WGS) further demonstrated that a segment of 1q43-q44 was inserted at 14q23.1 in the unaffected mother, which indicated that the mother was a carrier of a 46,XX,ins(14;1)(q23.1;q43q44) insertional translocation. Moreover, Sanger sequencing was used to assist the mapping of the breakpoints and the final validation of those breakpoints. The breakpoint on chromosome 1 disrupted the *EFCAB2* gene in the first intron, and the breakpoint on chromosome 14 disrupted the *PRKCH* gene within the 12th intron. In addition, fluorescence in situ hybridization (FISH) further confirmed that the unaffected older sister of the proband carried the same karyotype as the mother.

**Conclusion:**

Here, we describe a rare family exhibiting pure 1q43-q44 deletion and duplication in two siblings caused by a maternal balanced insertional translocation. Our study demonstrates that WGS with a carefully designed analysis pipeline is a powerful tool for identifying cryptic genomic balanced translocations and mapping the breakpoints at the nucleotide level and could be an effective method for explaining the relationship between karyotype and phenotype.

**Electronic supplementary material:**

The online version of this article (10.1186/s13039-018-0371-7) contains supplementary material, which is available to authorized users.

## Background

The clinical phenotype of 1q43-q44 deletion or duplication is highly variable, due to the size of the segment and the number of genes involved. The phenotypic features of patients with 1q43-44 deletion include moderate to severe mental retardation, development retardation, microcephaly, corpus callosum dysplasia, epilepsy and dysmorphic features. In individuals with 1q43-q44 duplication, the most recognizable features are macrocephaly, mental retardation, epilepsy and mild malformation. Interstitial deletions of the long arm of chromosome 1 involving only the 1q43-q44 region have been reported in more than 80 patients, with most of these patients arising de novo [1–7]. A few individuals exhibiting pure 1q43-q44 interstitial duplication have been reported [8–15]. However, both pure 1q43-q44 deletion and duplication occurring in a family have not been reported.

Several cytogenetic and molecular techniques have been applied to detect the deletion or duplication of pathogenic copies, such as G-banding, fluorescence in situ hybridization (FISH) and chromosomal microarrays (CMAs). However, these techniques present individual limitations and can often be technically challenging. Recent studies have shown that whole-genome sequencing (WGS) with a carefully designed data analysis pipeline is a more powerful tool for detecting chromosomal abnormalities due to its higher resolution and the ability to detect balanced translocations and small imbalances that cannot be detected with CMAs [16].

Insertional translocations are complex chromosomal rearrangements that require at least three breakpoints in the involved chromosome, with an incidence of 1:80,000 in live births [17]. Insertional translocations can be divided into simple intrachromosomal or interchromosomal insertional translocations and complex chromosomal insertional translocations [18]. Nowakowska et al. [19] found that 2.1% of de novo copy number variations (CNVs) are actually inherited from a parental balanced insertional translocation. However, this percentage may represent an underestimate because not all parental data may be collected in these studies, and due to technical limitations, some small imbalances have not yet to be discovered. However, WGS can identify nearly all cryptic chromosomal abnormalities or complex rearrangements present in the genome, in addition to characterizing translocation breakpoints at the nucleotide level.

Herein, we present a rare family in which two siblings presented with congenital anomalies. These two individuals harboured an approximately 6.0-Mb deletion or duplication of 1q43-q44 inherited from their mother, a carrier of a cryptic balanced insertional translocation. We further precisely identified the corresponding breakpoints via WGS and Sanger sequencing. This is the first report of the detection of an insertional translocation associated with 1q43-q44 deletion and duplication using WGS.

## Case presentation

**Fig. 1:**
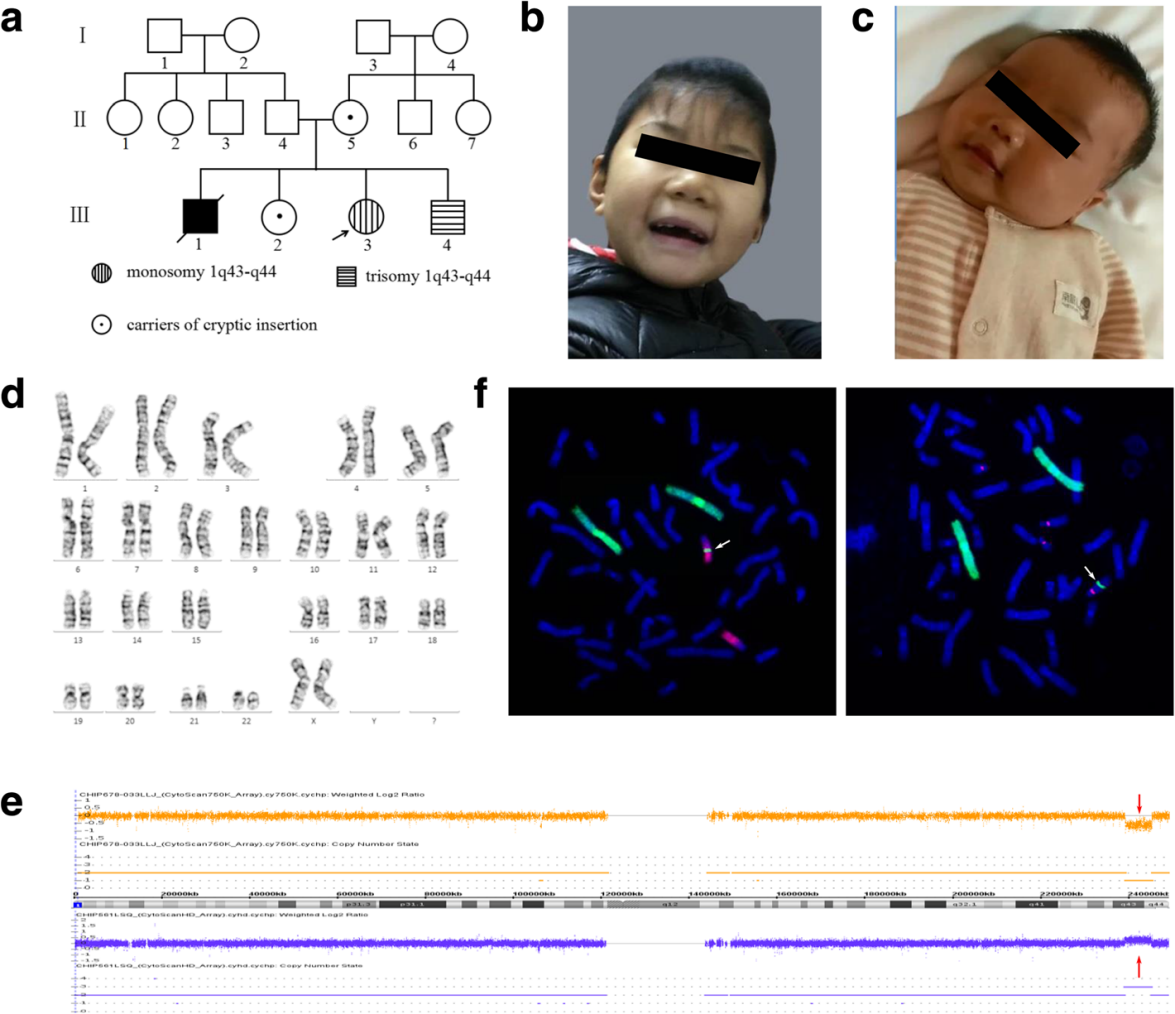
**a** Three-generation pedigree of the family with the proband (III-3) indicated by an arrow. Affected individuals are indicated with black, horizontal or vertical lines in the symbols, including III-3: monosomy 1q43-q44 and III-4: trisomy 1q43-q4; carriers of the cryptic insertion (II-5, III-2) are indicated with dots in the symbols. G-banding analysis was not performed for the elder brother (III-1) due to a lack of sample. **b** and **c** Facial profiles of the proband and younger brother at 6 years and 3 months, respectively. The proband presented with microcephaly, and the younger brother presented with macrocephaly. **d** G-banding analysis of the proband (III-3) at a band resolution of ∼400 showed no visual abnormal karyotype. **e** The results of SNP-array analysis. The proband (III-3) harbuored an interstitial 1q43-q44 deletion (upper), and the younger brother (III-4) carried an interstitial 1q43-q44 duplication (down). The deletion and duplication regions are indicated by red arrows. **f** The FISH results for the mother (II-5) with WCP1/14 (left) and WCP1/CEP14 (right) probes. Chromosome 1 is shown in green, and the chromosome 14 is shown in red (left); WCP1 and CEP14 are shown in green and red, respectively (right). The inserted segment is indicated by the white arrows

The proband (III-3, Fig. 1a) is the third child of a non-consanguineous, healthy couple. She is a 6-year-old Chinese girl with profound developmental delay, microcephaly, agenesis of the corpus callosum, epilepsy, language delayed and hearing impairment. She was born at full term after an uncomplicated spontaneous vaginal delivery with a normal birth weight (3400 g). She experienced seizures four times at 3 months of age, with spontaneous remission occurring after more than 10 s. At 7 months of age, she began turning over but could not grasp and sit without support. Intellectual evaluation with the Gesell Development Schedule (GDS) showed that her developmental quotient at 7 months of age was equivalent to that of a 10-week-old infant, indicating significant growth retardation [20]. The detailed data are shown in Additional file 1: Table S1. Brain magnetic resonance imaging (MRI) indicated absence of the corpus callosum and enlargement of the posterior horn of all three ventricles bilaterally. The results of brainstem auditory evoked potential (BAEP) analysis indicated bilateral hearing impairment. At 6 years of age, the proband presented with microcephaly (47.2 cm, <− 2 SD) and began learning to walk but could not speak (Fig. 1b).

The elder brother (III-1, Fig. 1a) presented similar features to the proband, such as developmental delay, cerebral palsy and intracranial haemorrhage after birth, and died at 5 years of age. The elder sister (III-2, Fig. 1a) has a normal phenotype. The younger brother (III-4, Fig. 1a) is the fourth child. At 34 weeks of gestation, an MRI scan of the foetal head and ultrasonography revealed no obvious abnormalities. He was born at term via caesarean section after an uneventful pregnancy and exhibited a normal birth weight (3000 g). At the age of 3 months, he presented macrocephaly (Fig. 1c) and his head circumference was 44.5 cm (> + 2 SD). His developmental quotient was equivalent to that of an 11-week-old infant, with testing demonstrating a borderline full-scale developmental quotient (85), and he exhibited developmental delay in gross motor functions (Additional file 1: Table S1).

## Materials and methods

G-banding at a band resolution of ∼400 was performed on metaphase peripheral blood lymphocytes obtained from the proband (III-3) and four other family members (II-4, II-5, III-2 and III-4) according to the laboratory’s protocols. DNA was isolated from their peripheral blood lymphocytes using the QIAamp® DNA blood midi kit (QIAGEN, Hilden, Germany). Single-nucleotide polymorphism (SNP) array analysis was performed using Cytoscan 750 K chips (Affymetrix, Santa Clara, CA, USA) as we described in a previous report [21]. The data were analysed using ChAS chromosome analysis software (Affymetrix, Santa Clara, CA, USA). To confirm the chromosomal imbalances of the patients and determine whether they were de novo or inherited from the parents, the parental DNA was evaluated by whole-genome low-coverage sequencing. Briefly, a non-size selected mate-pair library was prepared using ~ 3 μg of genomic DNA and then subjected to 50-bp-end multiplex sequencing on the Illumina HiSeq™ X10 platform. After automatically removing adaptor sequences and low-quality reads, high quality paired-end reads were aligned to the NCBI human reference genome (GRCh37/hg19) by SOAP2. Uniquely mapped reads were selected for subsequent analysis as previously described in detail [22]. After the bioinformatics analysis, we obtained the candidate breakpoint regions. The precise breakpoints were further confirmed by PCR and Sanger sequencing, and the genomic locations of the breakpoints were analysed according to the February 2009 (GRCh37/hg19) assembly in the UCSC Genome Browser (http://genome.ucsc.edu). Primers targeting the flanking sequences of the candidate breakpoints of chromosomes 1 and 14 were designed with Primer 5 software and are listed in Additional file 2: Table S2. To validate the abnormal karyotype, FISH was performed on metaphase chromosomes of peripheral blood lymphocytes using whole chromosome probes (WCPs) of chromosomes 1and 14 and a centromere probe (CEP) of chromosome 14 (CytoTrend, HK, China) following the manufacturer’s instructions. The chromosomes 14 and 22 with homologous regions in the centromeres were distinguished based on their different lengths.

## Results

G-banding analysis at a band resolution of ∼400 revealed no karyotype abnormalities in the proband (Fig. 1d) or the four other family members. However, further SNP array analysis indicated pathological CNVs in the proband and her younger brother: arr[hg19]1q43q44 (239,019,924–245,142,519) × 1 and arr[hg19]1q43q44(239,033,439–245,142,567) × 3, respectively (Fig. 1e).

**Fig. 2:**
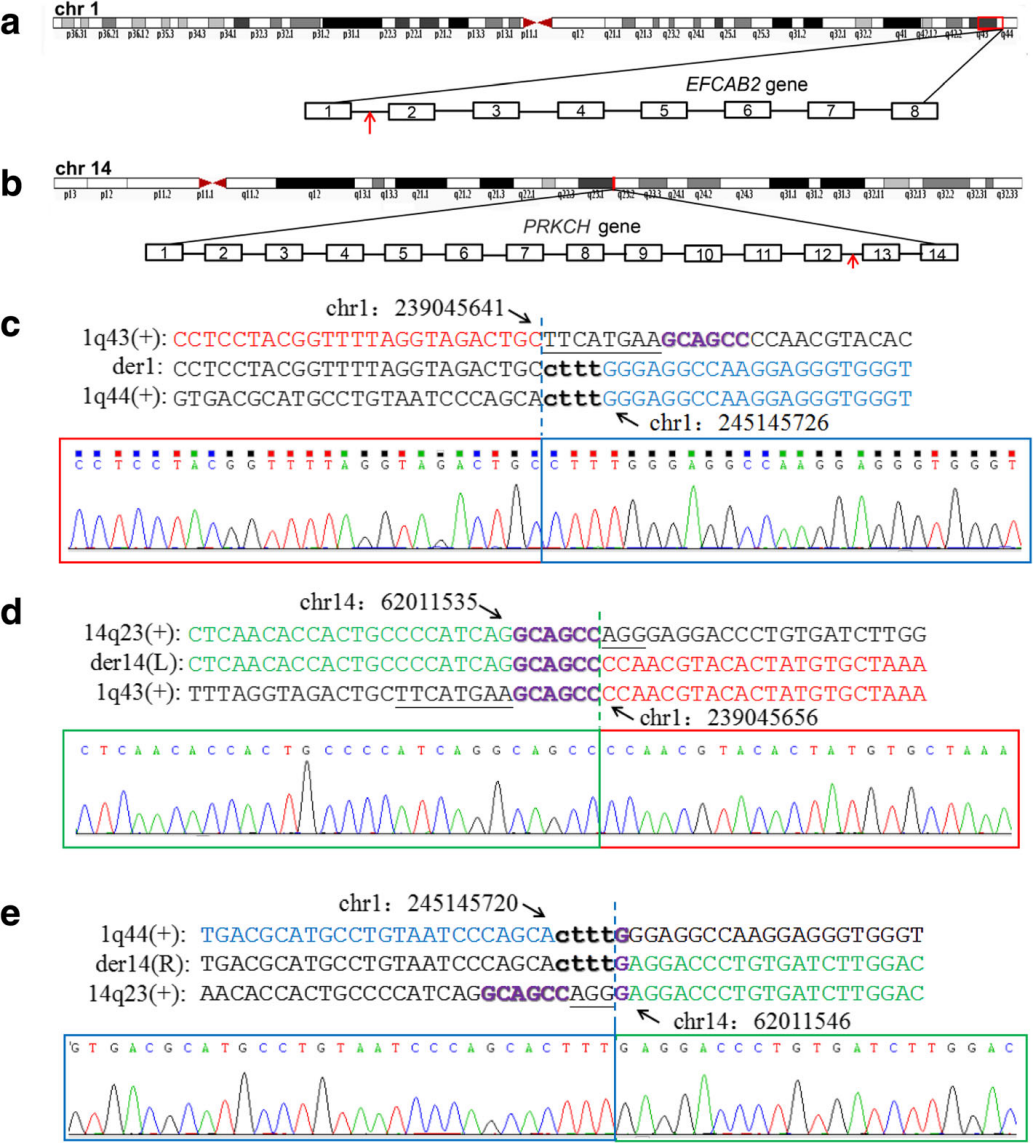
The chromosome breakpoints and disrupted genes within the insertional translocation t(1:14). **a** and **b** The disrupted genes at the breakpoints are indicated by red arrows. The breakpoint on chromosome 1q44 disrupts the *EFCAB2* gene in intron 1, and the breakpoint on chromosome 14 disrupts the *PRKCH* gene in intron 12. **c**-**e** The breakpoints mapped at the base-pair level by Sanger sequencing. Translocation junction sequences (middle line) and matching reference sequences (top and bottom lines) are shown with different colours depending on the involved chromosome region (1q43-red, 1q44-blue, 14q23-green). The microhomology observed at the translocation breakpoint sites are indicated in purple letters in bold, deleted sequences are underlined, and duplicate sequences are shown in lower-case letters in bold. Der14(L) indicates the breakpoint sequence near the centromere, and der14(R) indicates the breakpoint sequence near telomere

WGS analysis of the parents (II-4 and II-5) revealed a normal karyotype for the father but misalignment ~ 3.78 million reads for the mother. Further analysis showed that two records were highly credible (*p* < 0.001), roughly described as chr14-chr1:62,011,989–245,138,646 and chr14-chr1:62,006,695–239,045,980. These abnormal records indicated insertion of the 1q43-q44 segment into 14q23.1 in the mother’s genome, which was confirmed via FISH using the WCP1/14 and WCP1/CEP14 probes (Fig. 1f). The combination of WGS and FISH analyses revealed that the mother exhibited a 46,XX,ins(14;1)(q23.1;q43q44) karyotype. The three breakpoints were further determined by PCR and Sanger sequencing. Sanger sequencing further confirmed that the first breakpoint on chromosome 1 was located at chr1:239,045,641–239,046,656, the second breakpoint on chromosome 1 was located at chr1:245,145,720–245,145,726, and the breakpoint on chromosome 14 was located at chr14:62,011,535–62,011,546. There were no genes around the 1st breakpoint on chromosome 1. By contrast, the *EFCAB2* gene was disrupted in the first intron by the 2nd breakpoint on chromosome 1, and the breakpoint on chromosome 14 disrupted the *PRKCH* gene within the 12th intron (Fig. 2a and b). Moreover, some small imbalances and microhomology sequences were also observed near these breakpoint sites (Fig. 2c-e).

Further family analysis by G-banding and FISH confirmed that the elder sister carries the same balanced insertional translocation as the mother.

## Discussion

Here, we report a rare family in which two siblings exhibit 1q43-q44 deletion or duplication, respectively. The combination of SNP array, WGS and FISH analyses showed that both the deletion and duplication resulted from a 6.0-Mb cryptic balanced insertion of material from 1q43-q44 inserted into 14q23.1.

Chromosome 1q43-q44 deletion syndrome (OMIM: #612377) is characterized by moderate to severe mental retardation, limited or no speech, and variable but characteristic facial features, including a round face, prominent forehead, flat nasal bridge, hypertelorism, epicanthal folds, and low-set ears. Other characteristics may include developmental retardation, microcephaly, agenesis of the corpus callosum, and seizures [2]. Compared with 1q43-q44 deletion, the clinical manifestations of patients with 1q43-q44 duplication may be mild and mainly include macrocephaly, mental retardation and mild malformation [8, 23]. The clinical features of previously described patients with deletion or duplication of chromosome 1 overlapping q34-q44 are shown in Table 1 for a comparison of phenotypic differences. In the present study, the proband exhibiting 1q43-q44 deletion was found to show some characteristic features, such as profound developmental delay, microcephaly, agenesis of the corpus callosum, epilepsy, and unusual hearing impairment. The younger brother carrying this duplicated region presented with macrocephaly and mild developmental delay in gross motor functions. We speculate that some symptoms have not yet emerged in this child because he is very young or that other symptoms might be very mild.

**Table 1:** Features presented in patients with 1q43-q44 deletion and duplication

Clinical features	Part or all 1q43-q44 deletion [23]	This paper (proband)	Part or all 1q43-q44 duplication [8–14]	This paper (younger brother)
Microcephaly	61/81	+	1/8	–
macrocephaly	–	–	5/8	+
Intellectual disability	63/81	+	7/8	+
Corpus colllosum abnormalities	48/81	+	–	–
Seizures	52/81	+	4/8	–
Round face	16/81	–	–	–
Hypertelorism	14/81	–	5/8	–
Prominent forhead	10/81	–	6/8	–
Up or downward palpebral fissures	18/81	–	4/8	–
Long philtrum	18/81	–	3/8	–
Abnormal ear shape	29/81	–	6/8	–
Caradiac abnormalities	23/81	–	4/8	–
Abnormal hand or feet	25/81	–	6/8	–
Micrognathia	21/81	–	3/8	–
Hypotonia	43/81	–	2/8	–

“+”: present; “-”: absent

Many patients with 1q43-q44 deletion or duplication including part or all of the regions identified in our patients have been reported. However, the identification of well-detailed genotype-phenotype correlations is hindered by inaccurate mapping of the detailed breakpoints, due to the use of karyotyping or FISH analyses, before the era of high-resolution cytogenetics and the fact that those patients exhibit affected 1q43-q44 regions that differ in size and location. We identified a cryptic chromosomal rearrangement in the mother (II-5) via WGS and confirmed it via FISH. Moreover, we accurately mapped the breakpoints with a combination of a carefully designed data analysis pipeline and Sanger sequencing. The combination of these molecular and cytogenetics techniques characterized the breakpoints at the base-pair level and identified two intron-disrupted genes, *EFCAB2* and *PRKCH*, that have observable clinical phenotypes.

In our study, the two patients with 1q43-q44 deletion or duplication presented congenital anomalies, and the proband exhibited a more serious phenotype than her younger brother. Thus, dosage effects or pathogenic variants of some genes within 1q43-q44 likely contribute to their phenotypes. There are 20 known genes that lie within the 6.0-Mb genomic region, 9 of which are indicated to be disease genes in the Online Mendelian Inheritance in Man (OMIM) database according to NCBI Map Viewer (https://www.ncbi.nlm.nih.gov/mapview). The details of these 9 OMIM disease genes and their clinical characteristics and inheritances are shown in Table 2. We analysed the dominant genes for potential dose-effect phenotypes. The *AKT3* gene encodes a serine-threonine kinase belonging to the protein kinase B family that is highly expressed in the brain tissue of humans and rodents [24]. The expression of this gene is significantly decreased in the brain and corpus callosum of *AKT3*-null mice [25], and some studies in humans and mice have demonstrated that *AKT3* plays an important role in controlling the sizes of cells and organs [26, 27]. Boland et al. [5] reported a patient with a 46,XY,t(1;13)(q44;q32) translocation who presented postnatal microcephaly and agenesis of the corpus callosum and demonstrated that *AKT3* was a candidate gene for these phenotypes. Another study showed that a critical region comprising *CEP170, SDCCAG8* and *AKT3* was associated with microcephaly [4]. However, among patients with 1q43-q44 duplication, macrocephaly is observed in patients exhibiting *AKT3* gene duplication [8]. The *ZBTB18* gene encodes a protein that acts as a transcriptional repressor of key pro-neurogenic genes. Xiang et al. [28] found that conditional knockout of the *ZBTB18* gene in the central nervous system resulted in microcephaly, reduced thickness of the cortex, agenesis of the corpus callosum, and cerebellar hypoplasia. Thus, *ZBTB18* was proposed as the most likely candidate gene for corpus callosum abnormalities [2, 29]. These studies support pathological roles of *AKT3* and *ZBTB18* in the 1q43-q44 region. Furthermore, our findings support the notion that *ATK3* is a dosage-effect gene that may explain microcephaly or macrocephaly in patients with 1q43-q44 deletion or duplication, including our proband and her younger brother. Some studies have indicated that *HNRNPU* plays an important role in the regulation of embryonic brain development, and genetic mutation of *HNRNPU* might cause epileptic encephalopathy and intellectual disability [30–33]. Therefore, the *HNRNPU* gene may contribute to the seizure phenotypes of patients harbouring 1q43-q44 microdeletions. Furthermore, Bhatti et al. [34] found that homozygosity of 1q43-q44 deletion might cause non-syndromic hearing impairment and deemed a region containing *CHLM, OPN3* and *MAP1LC3C* a new autosomal recessive non-syndromic hearing impairment locus. In the present study, the proband also showed bilateral hearing impairment, but it may have been caused by genes of unknown function or other pathogenic factors. The elder brother presented a similar phenotype to that of the proband, and we cannot rule out the possibility that he might have exhibited the same karyotype as the proband. Analysis of families harbouring translocations via WGS and the associated analysis strategy can help us to gain a better understanding of the relationship between phenotype and karyotype, in addition to providing evidence for genetic and reproductive counselling, which may be especially important for the unaffected mother and sister, who are carriers of the insertional translocation.

**Table 2:** OMIM genes deleted or duplicated in our patients, with related phenotypes and model of inheritance

Gene	OMIM	Phenotype	Inheritance
*CHRM3*	118,494	Prune belly syndrome	AR
*FMN2*	606,373	Mental retardation, autosomal recessive 47	AR
*GREM2*	608,832	Tooth agenesis, selective, 9	AD
*FH*	136,850	Fumarase deficiency Leiomyomatosis and renal cell cancer	AR AD
*SDCCAG8*	613,524	Bardet-Biedl syndrome 16 Senior-Loken syndrome 7	AR -
*AKT3*	611,223	Megalencephaly-polymicrogyria-polydactyly-hydrocephalus syndrome 2	AD
*ZBTB18*	608,433	Mental retardation, autosomal dominant 22	AD
*COX20*	614,698	Mitochondrial complex IV deficiency	AR
*HNRNPU*	602,869	Epileptic encephalopathy, early infantile, 54	AD

*AD* Autosomal dominant, *AR* Autosomal recessive

Accurate breakpoint mapping not only facilitates the elucidation of the relationship between phenotype and karyotype but also offers insights into the possible mechanisms involved in the generation of balanced translocations. In this study, the molecular characterization of the breakpoints showed that they occurred in homologous regions between two non-homologous chromosomes, in addition to demonstrating the presence of small imbalances around the breakpoint site. These findings suggest that the translocation was likely generated through microhomology-mediated repair (MHMR) of double-strand breaks.

## Conclusion

In summary, we report a rare family in which two siblings exhibit pure 1q43-q44 deletion or duplication caused by a maternal balanced insertional translocation. Our study demonstrated that WGS is a powerful tool that allows rapid and accurate mapping of translocation breakpoints at the nucleotide level and could provide useful information for genetic and reproductive counselling for balanced translocation carriers. In addition, the results may help us to better understand detailed karyotype-phenotype correlations, and investigate the possible mechanisms underlying the generation of translocations.

## Additional files

Additional file 1: **Table S1.** The Gesell Development Scale results of the patients. (DOCX 16 kb)

Additional file 2: **Table S2.** The list of primers for PCR amplification of chromosomal breakpoint regions. (DOCX 16 kb)

## Abbreviations

CNV: Copy number variation
FISH: Fluorescence in situ hybridization
PCR: Polymerase chain reaction
SNP: Single-nucleotide polymorphism
WGS: Whole-genome sequencing
