# Supplementary material for: Maternal interchromosomal insertional translocation leading to 1q43-q44 deletion and duplication in two siblings
Source: Mol Cytogenet. 2018 Apr 4;11:24. doi: 10.1186/s13039-018-0371-7 (PMC5883343; doi:10.1186/s13039-018-0371-7)
Supplement: Supplementary file 1 — Table S1. The Gesell Development Scale results of the patients. (DOCX 16 kb) [file 13039_2018_371_MOESM1_ESM.docx]

**Table S1** The Gesell Development Scale results of the patients

|  | Development age (DA) | Development quotient (DQ) |
| --- | --- | --- |
| Adaptation | 10W^a^/11W^b^ | 31^a^/85^b^ |
| Gross motor | 11W^a^/9W^b^ | 34^a^/70^b^ |
| Fine motor | 12W^a^/12W^b^ | 37^a^/93^b^ |
| Language | 14W^a^/8W^b^ | 44^a^/62^b^ |
| Social contact | 12W^a^/9W^b^ | 37^a^/70^b^ |
| Total | 10W^a^/11W^b^ | 31^a^/85^b^ |

^a^ was indicated the results of the proband;

^b^ was indicated the results of the younger brother.
