# Supplementary material for: Maternal interchromosomal insertional translocation leading to 1q43-q44 deletion and duplication in two siblings
Source: Mol Cytogenet. 2018 Apr 4;11:24. doi: 10.1186/s13039-018-0371-7 (PMC5883343; doi:10.1186/s13039-018-0371-7)
Supplement: Supplementary file 2 — Table S2. The list of primers for PCR amplification of chromosomal breakpoint regions. (DOCX 16 kb) [file 13039_2018_371_MOESM2_ESM.docx]

**Table S2** The list of primers for PCR amplification of chromosomal breakpoint regions

| Primer | Sequence (5’-3’) | Annealing Tm (℃) | Product length (bp) |
| --- | --- | --- | --- |
| Der(1)-F | TATAATCTTCCAGCATGCATTG | 56 | 323 bp |
| Der(1)-R | CTAGGCTGGTCTCACACTCCT |  |  |
| Der14(L)-F | CATCAGCCTCCCAGATTTG | 54 | 544 bp |
| Der14(L)-R | GGTATATATGCCTAGTTAAAATTTTTCCT |  |  |
| Der14(R*)-F | GAGACTCTATTTAAGGACAAGTTCTTAGG | 56 | 697 bp |
| Der14(R*)-R | CCAGCACAAATGCAGAGG |  |  |

Der: derivative chromosome F: forward primer R: reverse primer

L: breakpoint sequence near centromere R*: breakpiont sequence near telomere
